# Morpho-Physiological and Proteome Level Responses to Cadmium Stress in Sorghum

**DOI:** 10.1371/journal.pone.0150431

**Published:** 2016-02-26

**Authors:** Swapan Kumar Roy, Seong-Woo Cho, Soo Jeong Kwon, Abu Hena Mostafa Kamal, Sang-Woo Kim, Myeong-Won Oh, Moon-Soon Lee, Keun-Yook Chung, Zhanguo Xin, Sun-Hee Woo

**Affiliations:** 1 Department of Crop Science, Chungbuk National University, Cheong-ju, Korea; 2 Division of Rice Research, National Institute of Crop Science, Rural Development Administration, Suwon, Korea; 3 Department of Chemistry and Biochemistry, University of Texas at Arlington, Arlington, Texas, United States of America; 4 National Agrobiodiversity Center, National Academy of Agricultural Science, Rural Development Administration, Jeonju, Korea; 5 Department of Industrial Plant Science & Technology, Chungbuk National University, Cheong-ju, Korea; 6 Department of Environmental & Biological Chemistry, Chungbuk National University, Cheong-ju, Republic of Korea; 7 Plant Stress and Germplasm Development Unit, USDA-ARS, 3810 4th Street, Lubbock, TX, United States of America; Nanjing Agricultural University, CHINA

## Abstract

Cadmium (Cd) stress may cause serious morphological and physiological abnormalities in addition to altering the proteome in plants. The present study was performed to explore Cd-induced morpho-physiological alterations and their potential associated mechanisms in *Sorghum bicolor* leaves at the protein level. Ten-day-old sorghum seedlings were exposed to different concentrations (0, 100, and 150 μM) of CdCl_2_, and different morpho-physiological responses were recorded. The effects of Cd exposure on protein expression patterns in *S*. *bicolor* were investigated using two-dimensional gel electrophoresis (2-DE) in samples derived from the leaves of both control and Cd-treated seedlings. The observed morphological changes revealed that the plants treated with Cd displayed dramatically altered shoot lengths, fresh weights and relative water content. In addition, the concentration of Cd was markedly increased by treatment with Cd, and the amount of Cd taken up by the shoots was significantly and directly correlated with the applied concentration of Cd. Using the 2-DE method, a total of 33 differentially expressed protein spots were analyzed using MALDI-TOF/TOF MS. Of these, treatment with Cd resulted in significant increases in 15 proteins and decreases in 18 proteins. Major changes were absorbed in the levels of proteins known to be involved in carbohydrate metabolism, transcriptional regulation, translation and stress responses. Proteomic results revealed that Cd stress had an inhibitory effect on carbon fixation, ATP production and the regulation of protein synthesis. Our study provides insights into the integrated molecular mechanisms involved in responses to Cd and the effects of Cd on the growth and physiological characteristics of sorghum seedlings. We have aimed to provide a reference describing the mechanisms involved in heavy metal damage to plants.

## Introduction

Over many decades, the industrial revolution has resulted in the environment being extensively polluted with different toxic metals and metalloids [1]. Heavy metals are among the most widespread soil contaminants, and they can cause damage to plants by inducing alterations in major physiological and metabolic processes [2,3,4,5,6]. The effects of certain heavy metals, such as cadmium (Cd), copper (Cu), lead (Pb) and zinc (Zn), have become an important concern for plant scientists because of their harmful effects on soil-plants and other cellular systems in the environment [7].

Cd has become a major environmental problem in agricultural systems where Cd has been ranked no. 7 among the top 50 toxic substances [8].Cd is introduced into the environment via many anthropogenic routes including power stations, heating systems, metal-working industries, waste incinerators, urban traffic, cement factories and as a byproduct of phosphate fertilizers [9].

Although Cd is a non-essential element in plants, it is easily taken up by roots and can be loaded into the xylem for transport to the leaves. The accumulated Cd within the plant induces various symptoms of toxicity, such as chlorosis, wilting, reduced growth and cell death [10]. Cd may influence plant physiological and biochemical processes by affecting the concentrations and functions of nutrient minerals that would otherwise have protective roles against the toxic effects of Cd-induced stress [11]. In addition, as a heavy metal, Cd has an adverse effect on the successful growth and development of plant [12].

However, when plants are exposed to high levels of heavy metals, they generate reactive oxygen species (ROS) as a primary response, and the combination of these new ROS with the oxidative stress already present in plants could be an indirect consequence of heavy metal toxicity [13,14]. Moreover, plants have evolved mechanisms to adapt to adverse environmental heavy metal toxicity pollution. To this end, plants produce low molecular weight thiols such as glutathione (GSH), phytochelatin (PCs) and cysteine that have a high affinity for toxic metals [15]. These compounds are produced primarily to be targets for free Cd-ions and GSH in particular increases heavy metal tolerance in plant cells and protects the cells from the oxidative stress that is induced by heavy metals [16] by directly binding metals in the cytosol and vacuole. Furthermore, GSH serves as a key regulator of redox signaling by altering gene expression at both the transcriptional and the translational level [17].

*S*. *bicolor* L. is an important crop because it is widely used as a food, feed and energy crop. In addition, sorghum has shown promise as a cereal crop, because it has some non-food uses, particularly in the production of bioethanol [18]. Previous studies demonstrated that sorghum plants were able to accumulate large quantities of heavy metal in the shoots [19,20], as well as highly tolerant to metal pollution [21,22]. Nevertheless, the availability of the sorghum full genome sequence [23] makes it a reasonable C_4_ model plant, which could be combined with the C_3_ plant models Arabidopsis and rice, to study the gene products involved in adaptation to heavy metal stress. Proteomics techniques present high throughput and large scale methods that can be used for the exploration of proteins in a particular organism, tissue or cell at any given time [24,25,26]. Two-dimensional electrophoresis (2-DE) in combination with mass spectrometry, has paved the way toward identifying the differentially expressed proteins that are produced in responses to various stresses in sorghum [27].

A considerable number of proteomics studies have been performed in plants exposed to abiotic stress conditions, but studies that involve exposure to heavy metals have been limited. Recently, proteomic techniques have been applied in many model and hyperaccumulator plants to identify Cd-regulated proteins at the molecular level [28,29,30,31,32,33,34,35]. Additionally, these studies have revealed that most of the studied plants undergo common changes in several types of functional proteins that are involved in energy and carbohydrate metabolism [28,33,36,37,38,39,40], transcription and translation [33,39,41] and stress-related proteins [33,38,40]. However, no report has been published to describe Cd-stress in C_4_ plants using proteomics. During the past decade, only a few studies dealing with the morpho-physiology and interactive effects of a combination of heavy metals on plants have been published [42], and some of these investigations have prompted the development of bioremediation [43] and phytoremediation [44] strategies. In addition, some investigations have been conducted to investigate the accumulated levels of heavy metals in the soil [45] and the growth and physiological characteristics of plants [12].

Taking these previous studies into account, in the present investigation, a high-throughput proteome technique was used to identify the differentially expressed proteins that may potentially be induced by different levels of Cd stress and to the response mechanisms induced by injury resulting from exposure to Cd in *S*. *bicolor*. Leaves of sorghum seedlings subjected to different levels of Cd were used as the experimental materials, and leaf proteome was performed using a 2-DE approach. The differentially expressed proteins identified by MALDI-TOF/TOF MS were compared to investigate cellular responses and to shed light on the molecular mechanisms underlying Cd-stress.

## Materials and Methods

### Plant growth condition and cadmium treatment

Sorghum [*Sorghum bicolor* (L.) Moench] inbred line BTx623 (http://www.phytozome.net/sorghum), which is a parent for several mapping populations in sorghum and the genotype for sequencing the sorghum genome, is used to generate the mutant populations [18]. It was developed by Dr. Fred Miller at Texas A & M University and released by Texas A & M University, Texas Agricultural Experiment Station in 1977. The pedigree of BTx623 is (BTx3197*SC170-6-4-4)-7-3-1-3-2-1. The lines are resistant to downy mildew, insecticidal leaf burn and rust zonate leaf spot. The lines have longer and wider stigmatic areas. These lines have the characteristics of tropical adaption, which produce higher yields under the short day length and hot night temperatures. The lines will be useful in forage and sugar sorghum production because of their resistance to downy mildew and other foliage pathogens and because of their sweet juicy stems.

Seeds of *S*. *bicolor* L. (BTX 623) were surface-sterilized and placed in petri dishes containing two layers of filter papers that were moistened with de-ionized water. The seeds placed in petri dishes were then placed in a growth chamber and grown in a controlled environment at 25°C with a light intensity of 8000 Lux and 70% humidity. After 5 days, the seedlings were transplanted to vessel containing Hoagland solution [46]. The solution was aerated daily via air bubbling for 30 min and changed every 2 days.

For the Cd-treatment experiments, three replicates of seven seedlings each were included in both the control and Cd treatment groups. The 10-day-old plants were exposed to Hoagland nutrient solutions, supplemented with 0, 100, 150 μM CdCl_2_ and grown under the same controlled environmental conditions described above. After 5 days of Cd-treatment, the seedling leaves were harvested and morpho-physiological and proteomic analyses were performed.

### Growth parameters

Shoot lengths (cm), root lengths (cm), shoot fresh weights (g), and root fresh weights (g) were measured from each of the collected samples. The dry weight of the shoot and root (g) was recorded in calibration balance (Ohaus Corporation, Pine Brook, NJ USA) after they were dried in a force oven at 65°C for 72 hours as previously described [47].

### Relative water content measurement

To evaluate the water status during the cadmium stress period, the relative water content (RWC) was determined as previously described [47]. Briefly, the plants (three seedlings) were weighed (fresh weight, FW) and, saturated in water for 2 hours and then their turgid weights (TWs) were calculated. The samples were dried in an oven at 65°C for 72 hours and their dry weights (DWs) were then calculated. RWC was determined as follows;

RWC = (FW− DW)/(TW −DW) X 100

### Determination of cadmium accumulation levels in leaves

The levels of cadmium in the leaves and root tissues were determined as previously described [48]. Briefly, the leaves and root tissues were collected, washed with distilled water, and dried at 105°C for 48 hours. Then, the dried materials were ground into a powder. Approximately 50 mg of this powder was digested in 5 ml of HNO_3_ (48%, w/v) at 60°C for 48 hours. After diluting the solution with Milli-Q water (1:20), the cadmium in the solution was measured using inductively coupled plasma-optical emission spectrometry (ICP-OES; Optima 5300 V; Perkin-Elmer, Inc., USA). The concentrations of cadmium in the tissues were calculated as mg per kg dry weight.

### Ion concentration measurements

The ion concentrations in the leaf tissues were determined as previously described [49]. Briefly, a portion (0.5 g) of the leaves of a sorghum plant was placed in a micro-Kjeldahl flask and 5 ml H_2_SO_4_ was then added. Filter paper (no.6 or no.7) was used to quantitate the amount of Zn^2+^, Ca^2+^, Fe^2+^, and Cd^2+^. The concentrations of positive ions, including cadmium, in the leaves and roots of the plants were determined using inductively coupled plasma-optical emission spectrometry (ICP-OES; Optima 5300 V; Perkin-Elmer, Inc., USA).

### Physiological analysis using confocal microscopy

For confocal microscopy, sorghum leaves were carefully cut and collected in petri dishes (60 x 15 mm) containing double-distilled water. After rinsing, the leaves were stained with a 100 μM aqueous solution of Dithizone for 30 min. They were then washed twice with double-distilled water for 5 min each. Finally, they were placed on slides, mounted in mounting solution and observed using confocal microscopy (LSM 410; Carl Zeiss, Jena, Germany).

### Protein extraction

Proteins were extracted from the leaves of *S*. *bicolor* using the trichloroacetic acid (TCA)/acetone method as previously described [50], with minor modifications. Frozen leaf tissues were ground to a fine powder in liquid nitrogen and homogenized in ice-cold 10% trichloroacetic acid and 0.07% 2-mercaptoethanol in acetone. After the suspension was vortexed, it was sonicated for 10 min, incubated for 1 hour at -20°C and centrifuged at 9000 x g for 20 min at 4°C. The supernatant was discarded and the pellet was washed twice with 0.07% 2-mercaptoethanol in acetone. The washed pellet was dried using a speed-Vac concentrator (Hanil Science Medical, Modulspin 31, Seoul, South Korea) for 10 min, resuspended in lysis buffer (8 M urea, 2 M thiourea, 5% CHAPS, and 2 mM tributylphosphine) with vortexing, and then incubated for 1 hour at 25°C. The suspension was then centrifuged at 20000 x g for 20 min at 25°C, and the resulting supernatant containing the protein extract was collected in a 1.5 ml tube. The protein concentrations of the samples were determined using the Bradford method [51], with a spectrophotometer (UV-1700 PharmaSpec; Shimadzu Corporation, Kyoto, Japan) using bovine serum albumin (BSA) as standard.

### 2-DE separation

The extracted protein samples were purified using a 2D cleanup kit (GE Healthcare Biosciences Corp, 800 Centennial Ave, Piscataway, USA). Protein extracts (100 μg) were suspended in rehydration buffer containing 8 M urea, 2% CHAPS, 50 mM DTT, 0.2% Bio-Lyte 3/10 ampholyte, and 0.001% Bromophenol Blue (Bio-Rad, Hercules, CA, USA) to attain a final volume of 150 μL. The solutions were directly loaded onto a focusing tray. Iso-electric focusing (IEF) was performed using immobilized pH gradient strips (3–10 NL, 7 cm Bio-Rad, USA) and then actively rehydrated for 12 hours at 50v. IEF was performed using a Protean IEF cell system (Bio-Rad) under the following sequential conditions; 250 V for 15 min with a linear ramp, 4000 V for 1 hour with a linear ramp, and 4000 V at 12000 V/h with a rapid ramp at 20°C. After IEF separation, the strips were incubated in 2 ml of equilibrium I (6 M urea, 2% w/v SDS, 0.375 M Tris- HCl (pH 8.8), 20% v/v glycerol and 2% w/v DTT) for 15 min and then equilibrated again for 15 min in the same buffer described above but with DTT replaced by iodoacetamide (2.5% w/v). For second-dimension electrophoresis, the equilibrated strips were transferred to 12% SDS-polyacrylamide gels with 5% stacking gels sealed with 1% agarose. Electrophoresis (2D) was performed at 25 V for the first 30 min and then 50 V until the bromophenol blue dye reached the bottom of the gel.

### Protein visualization and gel-image analysis

Gels obtained from 2-D PAGE were stained with Plus One Silver Staining Kit (GE Healthcare Biosciences AB, Uppsala, Sweden). The triplicate gels were scanned under HP Scanjet G 4010. The gel images were computationally analyzed using the Progenesis SameSpot software version 3.0 (Nonlinear Dynamics Ltd.). The intensity of all protein spots was normalized relative to the total abundance of all valid spots. After normalization and background subtraction, a matchset was created for both control gels (three replicates) and Cd-treated gels. To validate the automated spot detection and matching process, the images were edited manually and streaks, speckles, and artifacts were removed. Spot patterns of all gels were matched to each other to quantify each spot after normalization using the local regression model available in Progenesis SameSpots. The average intensities of resolved spots were compared using quantitative, qualitative and statistical functions within the Progenesis SameSpots software. Significant changes between spots were determined using Student's *t*-test for paired observations. Changes with a P value of < 0.05 were considered as being statistically significant.

### In gel digestion

To identify proteins separated by 2-DE, selected protein spots were manually excised from silver stained 2-DE gels. Then the gel slices washed with double distilled water were destained with 100 mM sodium thiosulfate and 30 mM potassium ferricyanide (1:1). The sample was then vortexed for 10 min, washed with distilled water for 3–5 times until completely destained and dehydrated for 10 min with 100% acetonitrile (ACN) and dried by vacuum centrifugation. After destaining, the gel pieces were reduced with 10 mM dithiothreitol in 100 mM NH_4_HCO_3_ for 1 hour at 56°C and again incubated with 55 mM iodoacetamide in 100 mM NH_4_HCO_3_ in the dark for 40 min. The gel slices were digested in 100 mM NH_4_HCO_3_ with 7–8 μL (0.1 μg/μL) trypsin enzyme (Promega Corporation, Madison, WI 53711–5399, USA) and incubated at 37°C for 16 hours. The tryptic peptides were extracted from the gel grains with 5% trifluoroacetic acid (TFA) in 50% acetonitrile 3 times. The solution containing eluted peptide was concentrated up to drying by vacuum centrifugation and the resultant extracts were analyzed by mass spectrometry.

### Protein identification by mass spectrometry

Differentially expressed protein spots were identified by matrix assisted laser desorption/ionization time-of-flight tandem mass spectrometry (MALDI-TOF/TOF MS). The peptides were eluted with 1μL matrix solution (α-cyano-4-hydroxy-cinnamic acid in 5% TFA, 50% acetonitrile) before application to the target plate. Samples were allowed to be air dry and analyzed by 4700 MALDI-TOF-TOF analyzer (Applied Biosymtems, Foster City, CA, USA). All mass spectra were acquired in the reflection mode with 0–4000 m/z by a 4700 proteomics analyzer (Applied Bio-systems, Framingham, MA, USA). External calibration was performed using a standard peptide mixture of des-Arg bradykinin, angiotensin, Glufibrinopeptide B, adrenocorticotropic hormone (ACTH) clip 1–17, ACTH clip 18–39, and ACTH clip 7–38.

### Bioinformatics analysis

The acquired MS and MS/MS spectra were used to identify differentially regulated proteins using Mascot Generic File (MGF) with an in-house licensed MASCOT search engine (Mascot v. 2.4.0, Matrix Science, London, UK) against the viridiplantae within the UniProt database. In the MASCOT search, carbamidomethylation of cysteines was set as a fixed modification and the oxidation of methionines was set as a variable modification. MASCOT was used with the monoisotopic mass selected, a peptide mass tolerance of 100 ppm, and a fragment iron mass tolerance of 2 Da. Trypsin was specified as the proteolytic enzyme with one potential missed cleavage. All proteins identified by high-scoring peptides were considered true matches, and at least two peptide matches. Protein hits were validated if the identification involved at least 10 top-ranking peptides with *p* < 0.05 and also selected false positive rate < 0.05. When those peptides matched multiple members of a protein family, the presented protein was selected based on the highest score with at least two peptide matching.

## Results

### Morphological responses of sorghum seedlings to Cd stress

**Fig 1:**
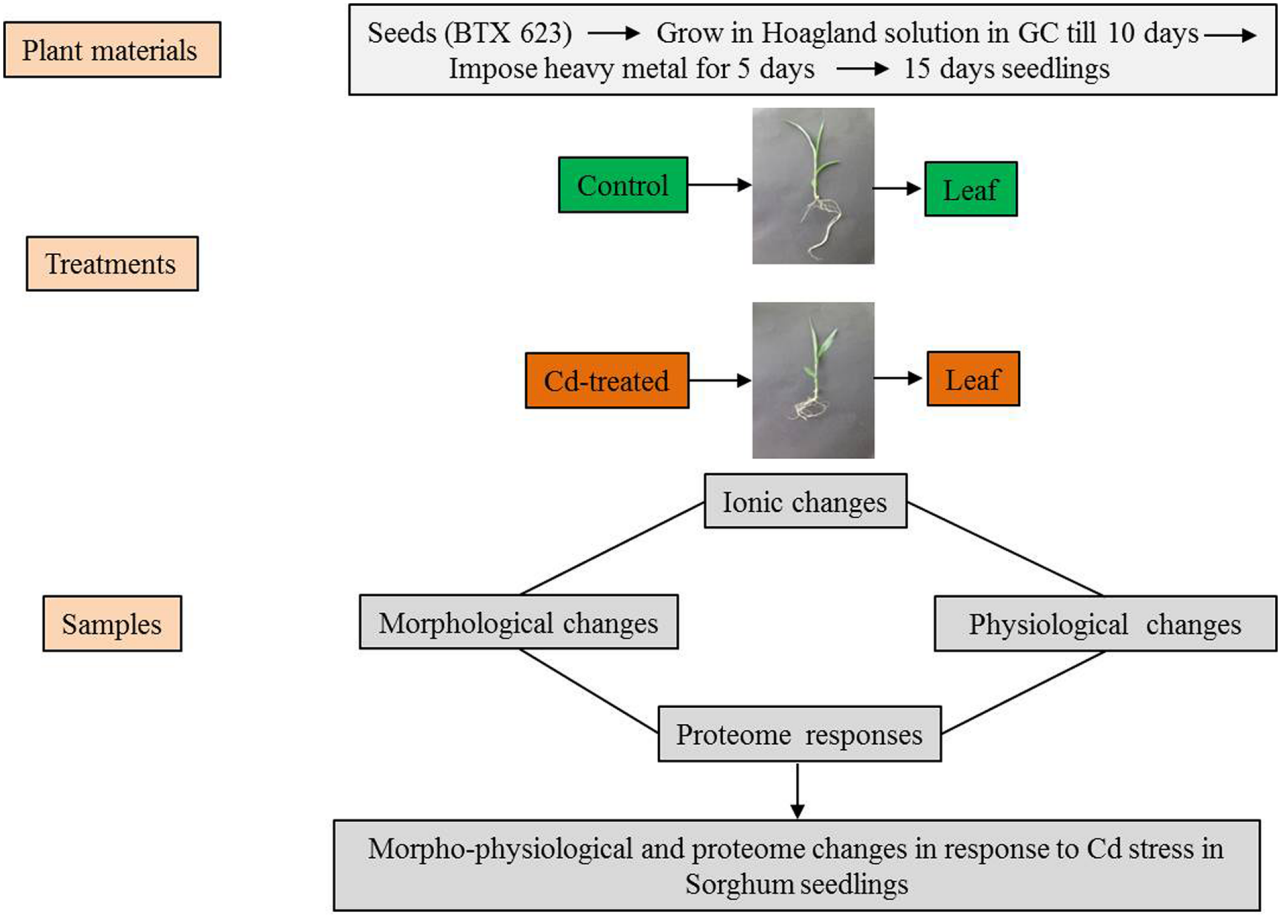
Schematic representation of the experimental setup used to compare Cd-treated sorghum seedlings with untreated plants (Control). The sorghum seedlings (BTX 623) were grown in growth chambers for 10 days in Hoagland solution. Ten-day-old sorghum seedlings were exposed to different concentrations of cadmium (0 μM, 100 μM, 150 μM CdCl_2_) for 5 days. The samples were collected from the control and Cd-induced leaves for measurement of morphological and physiological parameters (shoot lengths, fresh weights, relative water content, confocal and ion analysis). For proteome analysis, leaves were excised, pooled, rinsed with de-ionized water, rapidly frozen in liquid nitrogen, and stored at -80°C. Molecular changes were investigated in *S*. *bicolor* using two-dimensional gel electrophoresis (2-DE) in samples derived from the leaves of both control and Cd-treated seedlings.

**Fig 2:**
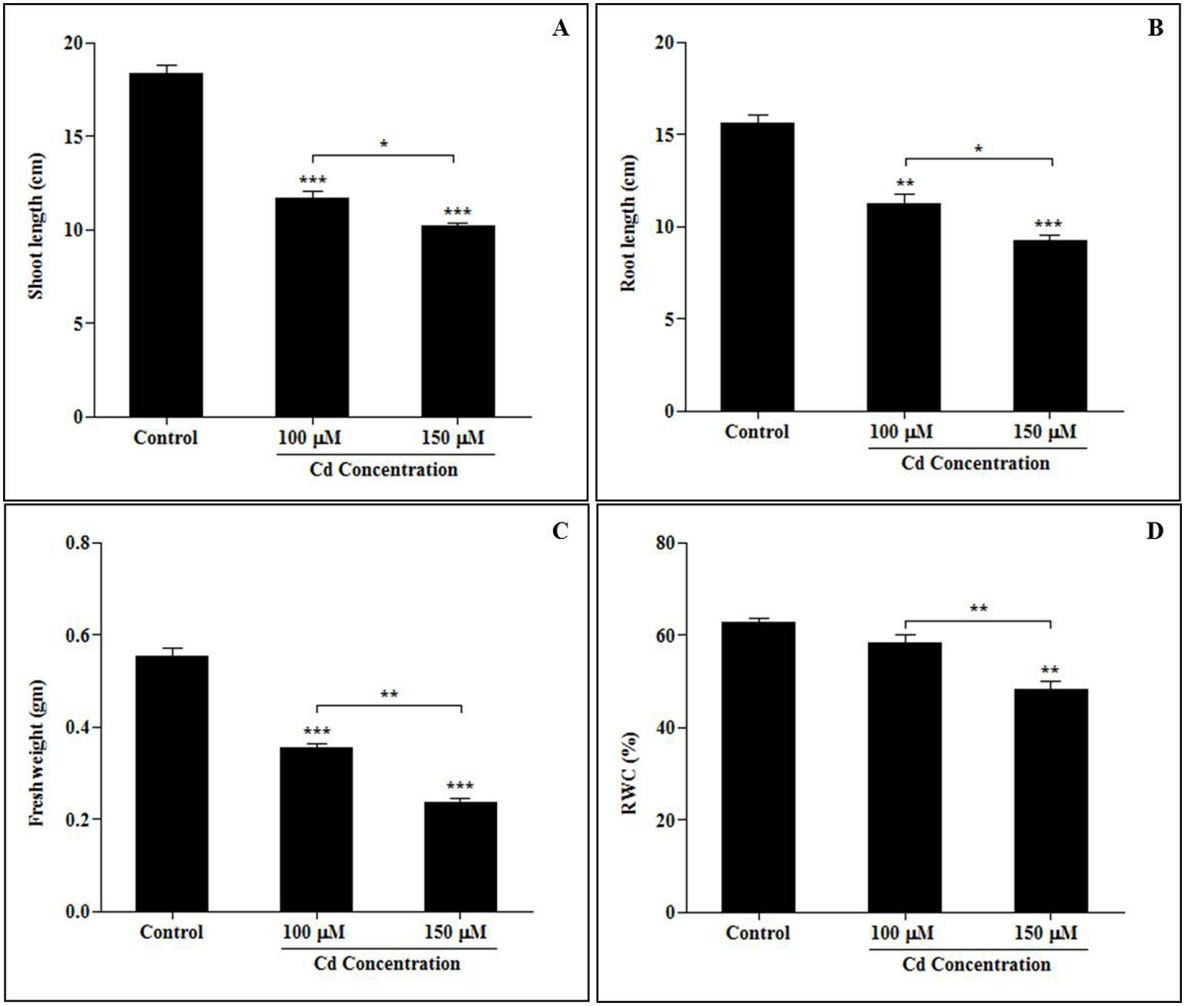
Responses induced by cadmium stress in morphological alterations in sorghum seedlings (A. Shoot length, B. Root length, C. Fresh weight, D. Relative water content) exposed to different concentrations of cadmium. Ten-day-old sorghum seedlings (BTX 623) were exposed to different concentrations of cadmium (0 μM, 100 μM, 150 μM CdCl_2_) for 5 days. After 5 days of Cd stress, the leaves and roots were collected from both the control and Cd-treated seedlings. Prior to measuring morphological and physiological parameters, the seedlings were washed with de-ionized water. Three plants were randomly selected for measurements at each time point for each replicate, and the experiment was biologically replicated 3 times. Each bar represents the average ± SE for 3 plants. Significant differences between the control and the cadmium-induced seedlings were determined by performing a one-way analysis of variance (ANOVA) with Tukey’s all pairs of column comparison test. Asterisks indicate significant differences at *p* < 0.05.

Our investigation was focused on studying the effects of exposing sorghum plants to different concentrations (0 μM, 100 μM and 150 μM) of CdCl_2_. The metal ions affected growth parameters and caused physiological alterations. These are schematically described in Fig 1. To understand the initial morphological responses induced in the sorghum seedlings by Cd stress, the length, fresh weight and relative water content (RWC) of their roots and shoots were measured at 15 days after planting (Fig 2). The growth and characteristics of the sorghum seedlings were analyzed following treatment with Cd, and morphological characteristics were found to be reduced by Cd stress (Fig 2). The most significant growth inhibition was observed in plants treated with the highest concentrations of Cd^2+^ ions (150 μM).

### Alterations in accumulated cadmium levels and ion concentrations following Cd treatment

**Fig 3:**
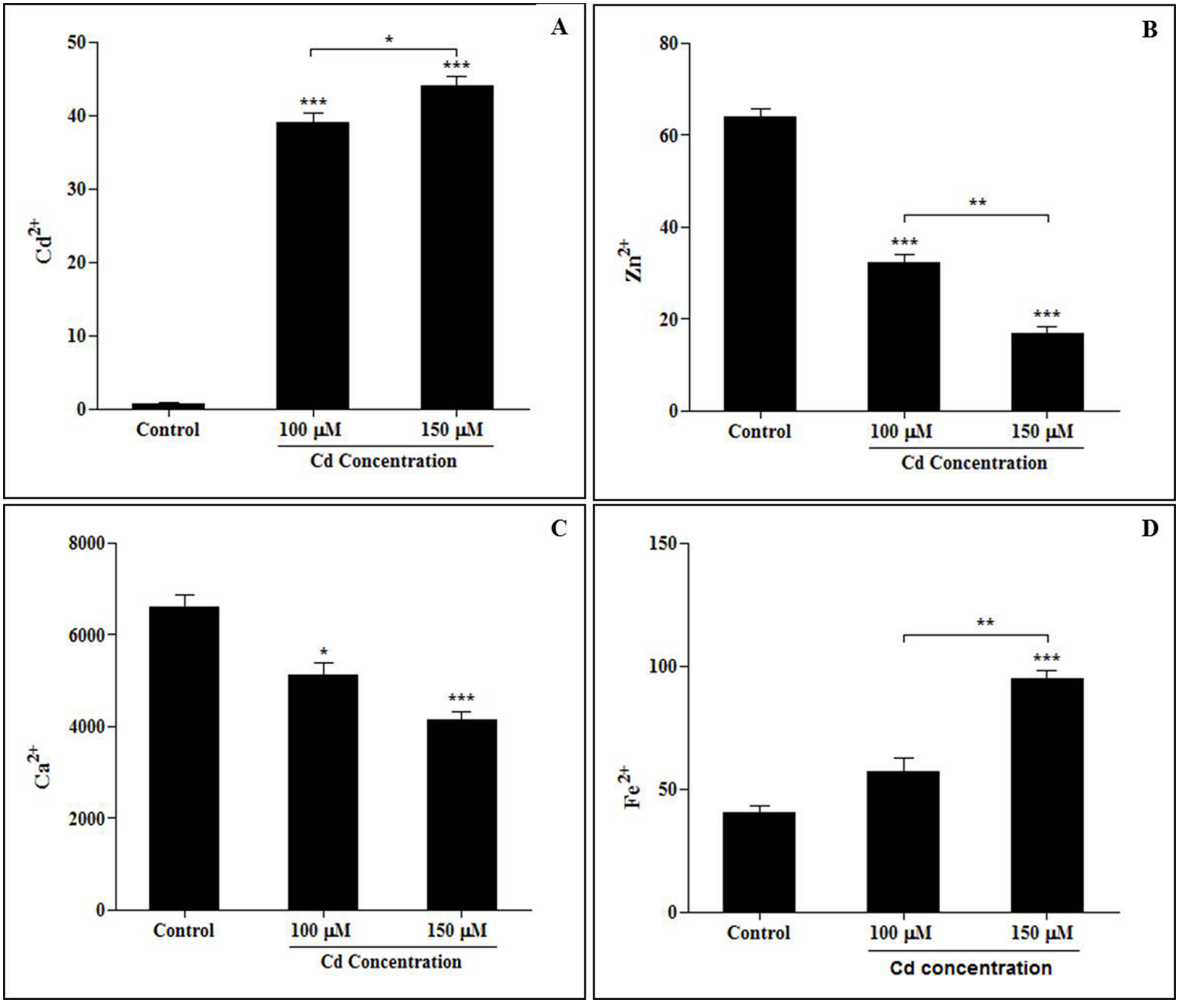
Ion concentrations and cadmium accumulation in sorghum leaves (A. Cd^2+^, B. Zn^2+^, C. Ca^2+^, and D. Fe^2+^) exposed to cadmium stress. Ten-day-old sorghum seedlings (BTX 623) were exposed to different concentrations of cadmium (0 μM, 100 μM, 150 μM CdCl_2_) for 5 days. Values (means ± SD) were determined for 3 independent experiments (n = 3).

Cadmium content was determined in the leaves of the experimental plants. High accumulated levels of Cd were observed in leaves of plants that were treated with different concentrations of Cd (Fig 3A–3D). We found that the accumulated level of Cd was significantly increased in plants exposed to the high concentration of Cd (150 μM) compared to the untreated plants (Fig 3A). In the present investigation, Cd^2+,^ Zn^2+^, Ca^2+^ and Fe^2+^ concentrations were determined in sorghum leaves. The concentrations of Zn^2+^ (Fig 3B) and Ca^2+^ (Fig 3C) ions were decreased, whereas the Fe^2+^ ion concentration was increased when seedling leaves were exposed to Cd^2+^ (Fig 3D). The concentration of cadmium in the plants was significantly increased by exposure to higher concentrations of cadmium.

### Effects of absorbed Cd ions as analyzed using confocal microscopy

**Fig 4:**
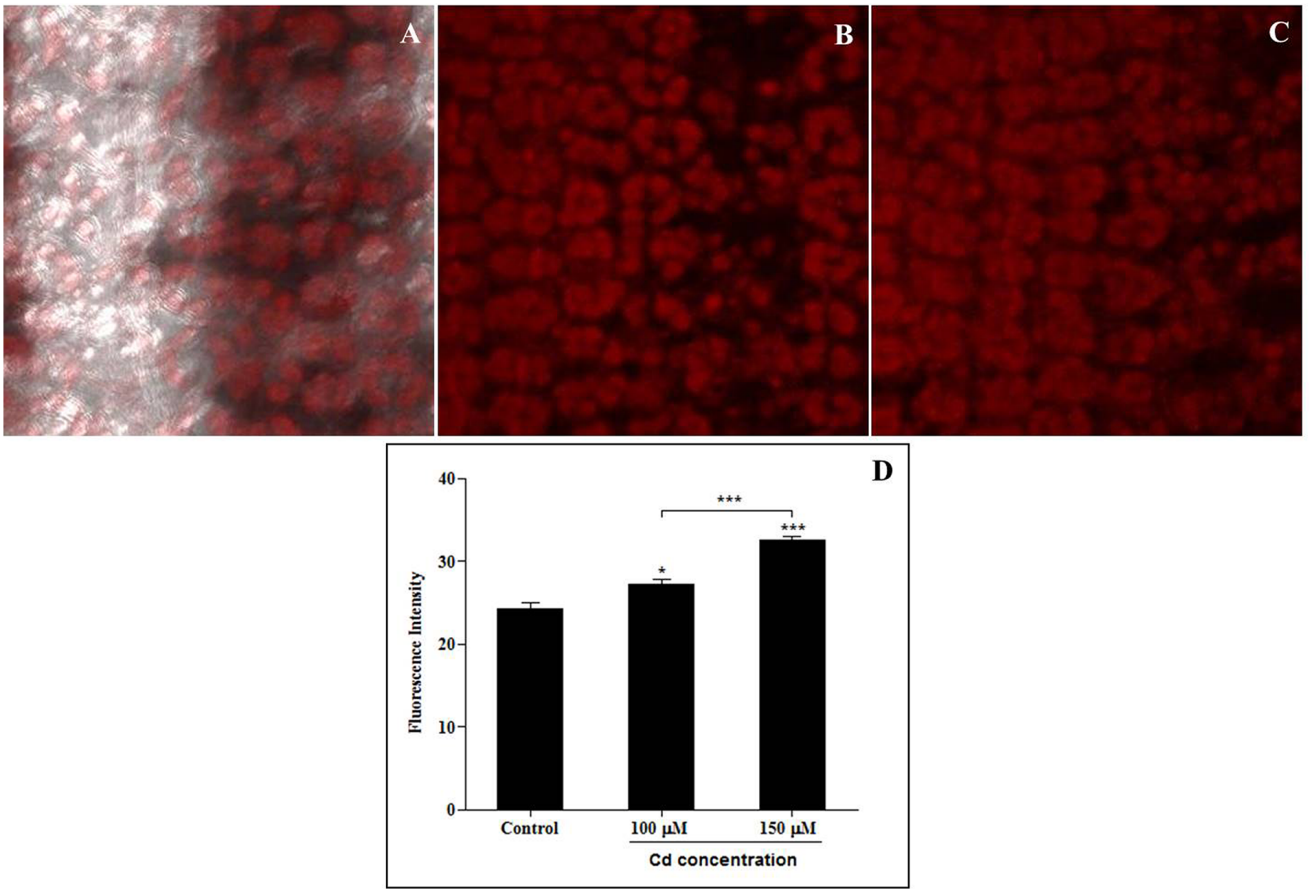
Cadmium distribution and fluorescence intensities in sorghum seedling leaves treated with dithizone staining. A. Control, B. 100 μM CdCl_2_, C. 150 μM CdCl_2_, D. Effect on the fluorescence intensity of cadmium-dithizone complexes. Samples obtained from different cadmium-treated leaves were placed on slides, mounted in mounting solution and observed using confocal microscopy (LSM 410; Carl Zeiss, Jena, Germany).

Confocal microscopy was used to analyze the distribution of Cd absorption in sorghum leaves. Each of the sorghum leaves was cut, stained with dithizone staining solution and then observed using confocal microscopy. Cadmium-dithizone complexes and fluorescence intensity were measured using confocal microscopy. The distribution of cadmium in the sorghum leaves, which was revealed by dithizone staining, is shown in Fig 4A–4D. We found that the degree of absorption of cadmium was increased by its concentration, as was the fluorescence intensity of Cd-dithizone (Fig 4D).

### Changes in proteomic expression patterns in sorghum seedling leaves in response to Cd

**Fig 5:**
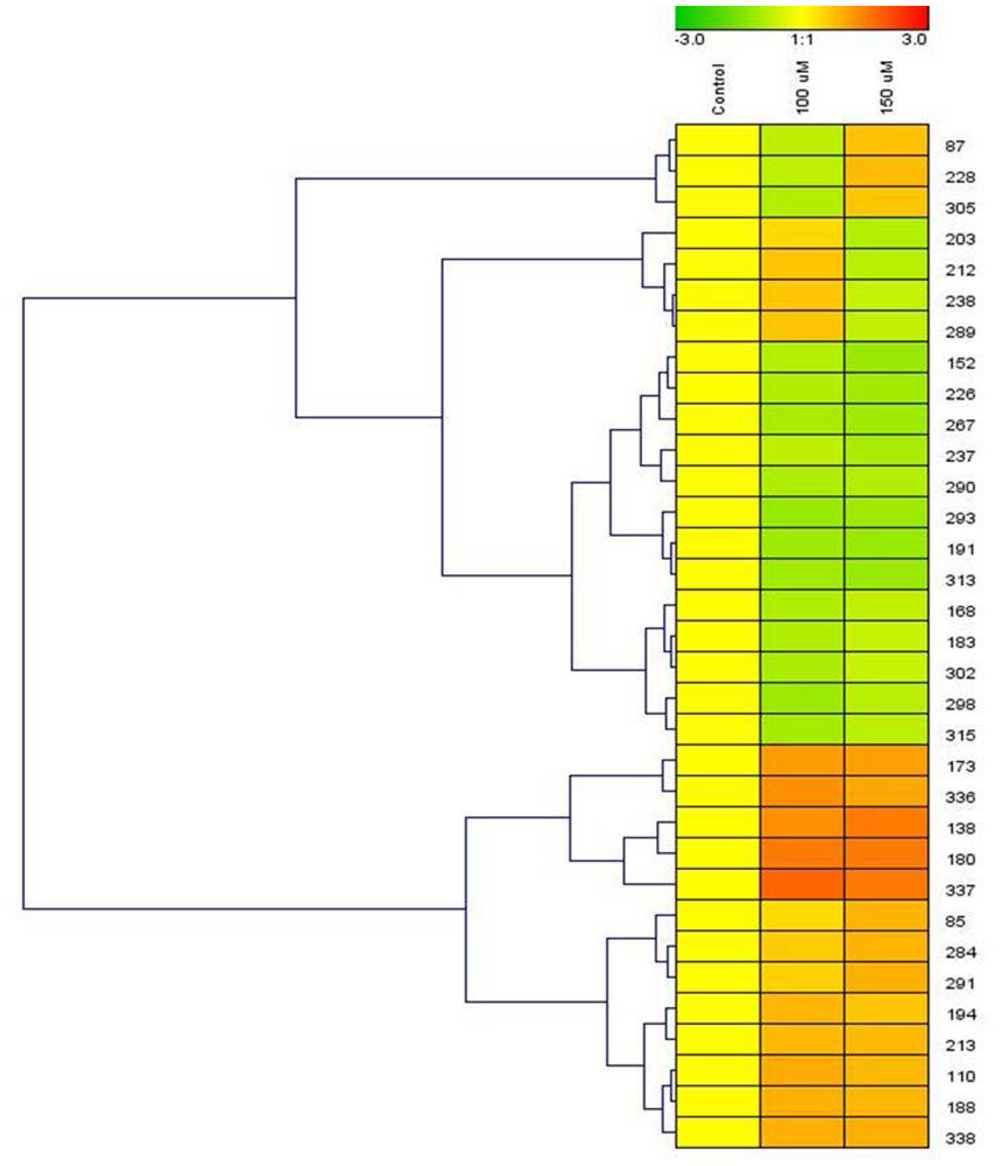
Cluster analysis of cadmium-responsive leaf proteins. Ten-day-old sorghum seedlings were exposed to cadmium stress for 5 days. Samples from non-treated control plants and treated plants were collected on the same day. Differences in the intensity of labeling associated with the proteins in both the control and the cadmium-treated samples are shown as clusters. Any statistically significant difference (*p* < 0.05) in labeling intensity was considered to be positive. The protein spot numbers are indicated on the right side of the cluster. The clusters were determined using Genesis software (ver. 1.7.6).

**Fig 6:**
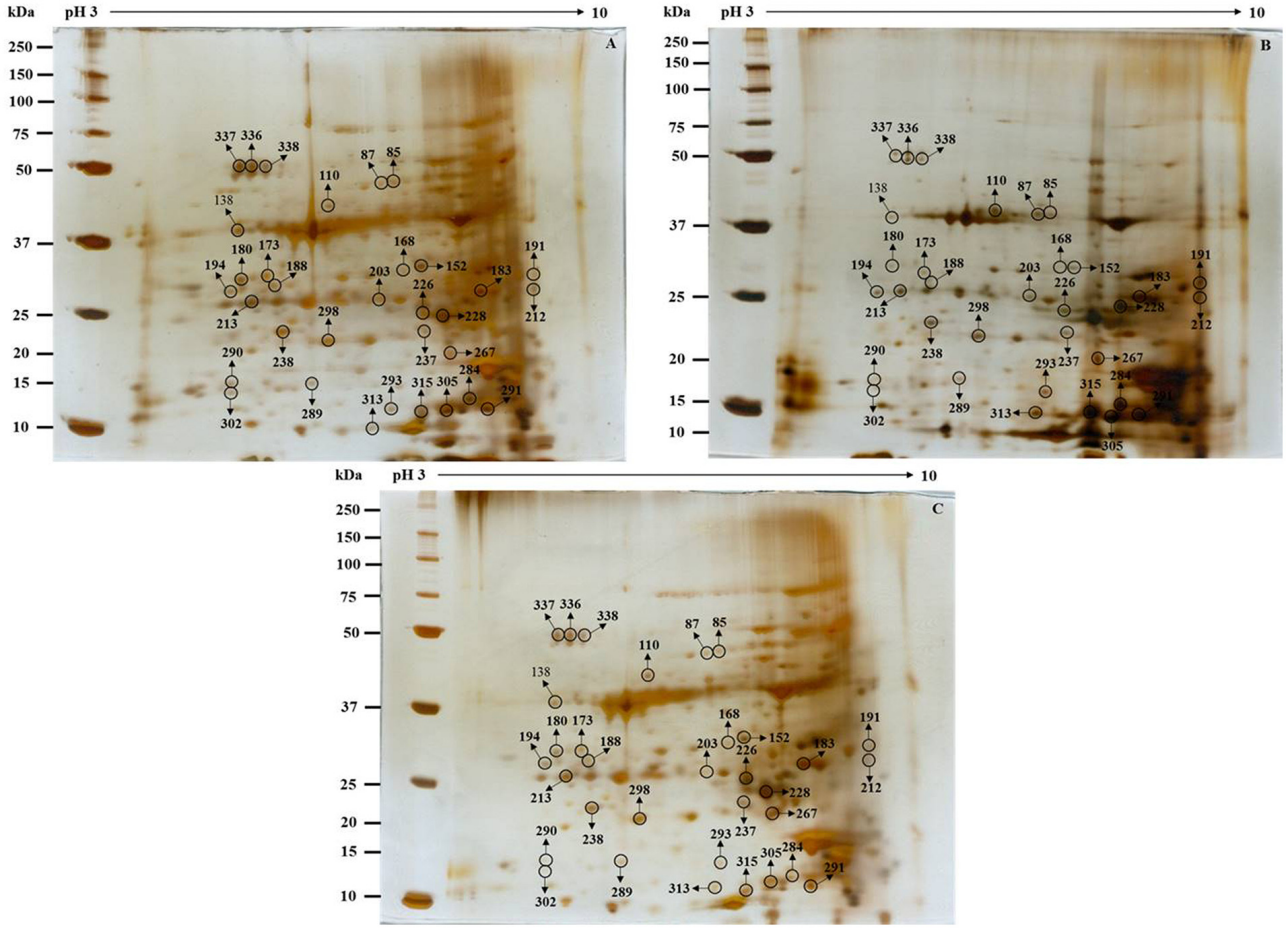
Representative images of gels used in 2-DE analysis of *S*. *bicolor* leaves exposed to 0 μM, 100 μM and 150 μM CdCl_2_. Leaf tissues were extracted using TCA-acetone precipitation method as described in the Materials and Methods section. Proteins were extracted from the leaves of 15-day-old seedlings that were treated with cadmium for 5 days. For IEF, 100 μg of proteins was loaded onto pH 3–10 NL IPG strips (7 cm). SDS-PAGE was performed on 12% gels, and the proteins were separated using 2-DE and then stained with silver staining. The differentially expressed protein spots (>1.5-fold difference) are indicated by circles on the 2-D gel map. These spots were found to be statistically significant at a level of 95% per group (Student’s *t*-test) using biological and analytical replicates (n = 3). The MW of each protein was determined using standard protein markers.

**Fig 7:**
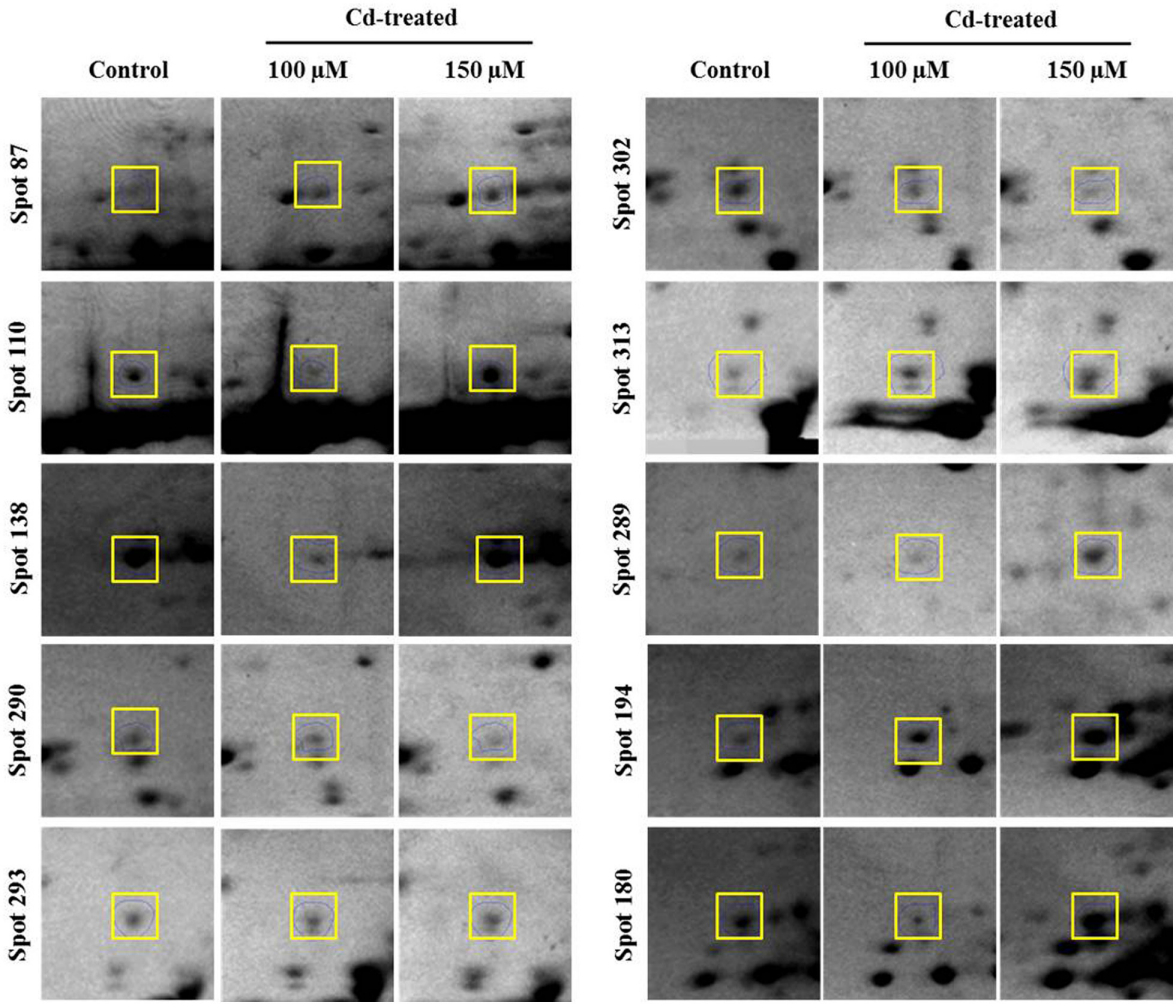
Magnified views of some of the differentially expressed protein spots that correspond to the identified proteins. The protein spots that were identified using mass spectrometry are indicated by squares and labeled in the figure.

To explore the underlying mechanisms involved in *S*. *bicolor* tolerance to Cd stress, the leaf proteome of *S*. *bicolor* samples was evaluated using two-dimensional electrophoresis. Total soluble proteins were extracted from the leaves of control (0 μmol CdCl_2_) and Cd-treated (100 μmol and 150 μmol CdCl_2_) sorghum seedlings. Each experiment was replicated three times, and more than 800 protein spots were reproducibly detected within each sample after silver staining based on our analysis of Progenesis SameSpot software. Quantitative image analysis revealed a total of 33 protein spots that exhibited more than a 1.5-fold changes in intensity (Fig 5). All spots of interests were located, manually excised, and subjected to in-gel tryptic digestion, and the 33 differentially expressed proteins were successfully analyzed using MALDI-TOF/TOF MS analysis. Among these proteins, a total of 15 proteins showed increased expression, and 18 proteins showed decreased expression in the treated samples compared to their levels in untreated seedlings (Fig 6). An enlarged image of a number of the identified gel spots is shown to illustrate the changes observed in the differentially expressed protein spots among the groups (Fig 7). The identified proteins are listed in Table 1.

**Table 1:** List of differentially identified proteins altered by Cd stress in the leaves of *S*. *bicolor* using MALDI-TOF-TOF mass spectrometry.

Spot Number^a^	Accession Number^b^	Protein Description	Protein Score^c^	Molecular Weight^d^ (kDa)	Protein Matches^e^	Protein Coverage^f^ (%)	Fold Change^g^	
							100 μM/Control	150 μM/Control
**Translation**								
85	RR2_OSTTA	30S ribosomal protein S2	48	25468	3	17	1.04↑	1.44↓
203	RPOA_PANGI	DNA-directed RNA polymerase subunit alpha	32	40481	2	18	1.07↑	1.47↓
213	RR3_ADICA	30S ribosomal protein S3	35	24952	3	28	1.71↑	1.33↓
238	RK5_MESVI	50S ribosomal protein L5, chloroplastic	32	20808	2	43	1.45↑	1.11↓
**Signal Transduction**								
87	CDPK1_ORYSJ	Calcium-dependent protein kinase isoform 1	56	61136	5	34	1.08↓	1.19↑
**Transcription**								
152	MATK_DIOEL	Maturase K	32	62406	2	15	1.25↓	1.89↓
183	MATK_NANDO	Maturase K	43	60828	3	25	1.33↓	1.06↓
194	MATK_ARCUU	Maturase K	36	59873	3	23	1.76↓	1.12↓
337	DRB1_ORYSJ	Double-stranded RNA-binding protein 1	37	47483	3	27	3.42↑	2.57↑
**Transport**								
110	PPI1_ARATH	Proton pump-interactor 1	46	68951	3	16	1.98↑	1.32↑
168	OEP37_ARATH	Outer envelope pore protein 37	51	38982	3	20	1.32↓	1.16↓
290	HMA2_ARATH	Cadmium/zinc-transporting ATPase	34	103806	5	14	1.35↓	1.47↓
293	PCR7_ARATH	Protein PLANT CADMIUM RESISTANCE 7	38	15620	4	19	1.79↓	1.79↓
**Transcriptional regulation**								
188	PP193_ARATH	Pentatricopeptide repeat-containing protein At2g38420	31	52248	2	57	1.89↑	1.36↑
191	C3H14_ARATH	Zinc finger CCCH domain-containing protein 14	37	35656	2	45	1.67↑	1.92↑
**Cell wall reorganization**								
237	CADH2_ARATH	Cinnamyl alcohol dehydrogenase 2	68	41339	2	21	1.03↓	1.61↓
**Stress response**								
173	C71AO_ARATH	Cytochrome P450	49	56426	4	25	2.29↑	1.83↑
180	C75A2_SOLME	Flavonoid 3',5'-hydroxylase	63	57974	6	39	2.97↑	2.53↑
228	GSTUO_ARATH	Glutathione S-transferase	61	25461	2	32	1.04↓	1.31↑
**Photosynthesis**								
305	PSBQ2_ARATH	Oxygen-evolving enhancer protein 3–2	75	24628	3	13	1.27↓	1.07↓
302	RBS1_ORYSI	Ribulose bisphosphate carboxylase small chain	59	19862	3	17	1.43↓	1.03↓
**Metabolism**								
**Cellular metabolism**								
212	CCNB2_MEDSA	G2/mitotic-specific cyclin-2	43	38142	6	47	1.48↑	1.32↑
**Energy and carbohydrate metabolism**								
226	ATPA_CERDE	ATP synthase subunit alpha	53	55230	2	24	1.30↓	1.75↓
**Alcohol metabolic process**								
267	ERG14_ARATH	Squalene epoxidase 1	35	58878	3	36	1.47↓	1.82↓
**Carbohydrate metabolism**								
313	SGGP_ARATH	Haloacid dehalogenase	82	26886	2	29	1.61↓	1.89↓
**Photosynthesis and carbohydrate metabolism**								
315	CAHC_SPIOL	Carbonic anhydrase, chloroplastic	39	34947	2	19	1.52↓	1.27↓
**Glycolysis and carbohydrate metabolism**								
338	G3P_ATRNU	Glyceraldehyde-3-phosphate dehydrogenase	49	39415	2	32	1.91↑	1.53↑
**Redox homeostasis and defense response**								
336	AAT3_ARATH	Aspartate aminotransferase 3, chloroplastic	38	49152	3	14	2.54↑	1.67↑
**Unknown**								
138	BRXL1_ARATH	Protein Brevis radix-like 1	67	38152	5	24	2.55↑	2.49↑
284	BMT_GLELI	Bergaptol O-methyltransferase	46	39815	4	31	1.36↑	1.41↑
289	CAPZB_ARATH	Probable F-actin-capping protein subunit beta	42	29200	6	31	1.45↑	1.14↑
291	CX5B2_ARATH	Cytochrome c oxidase subunit 5b-2	37	18856	5	15	1.23↑	1.49↑
298	SLSG0_BRAOA	S-locus-specific glycoprotein	45	51010	2	18	1.72↓	1.30↓

^a^ represents the spot number on the 2-DE gels shown in Fig 6

^b^ accession number according to the UniProt database

^c^ Score of the identified peptides

^d^ molecular weight of the identified protein

^e^ number of matched peptide, the proteins with >2 matched peptides were considered

^f^ protein coverage, the proteins with less than 10% sequence coverage was excluded from the result

^g^ fold change of protein quantities in cadmium treated sample against control samples

The symbol ↑ denotes the proteins were up-regulated and ↓ denotes the proteins were down-regulated.

### Functional classification of identified proteins

**Fig 8:**
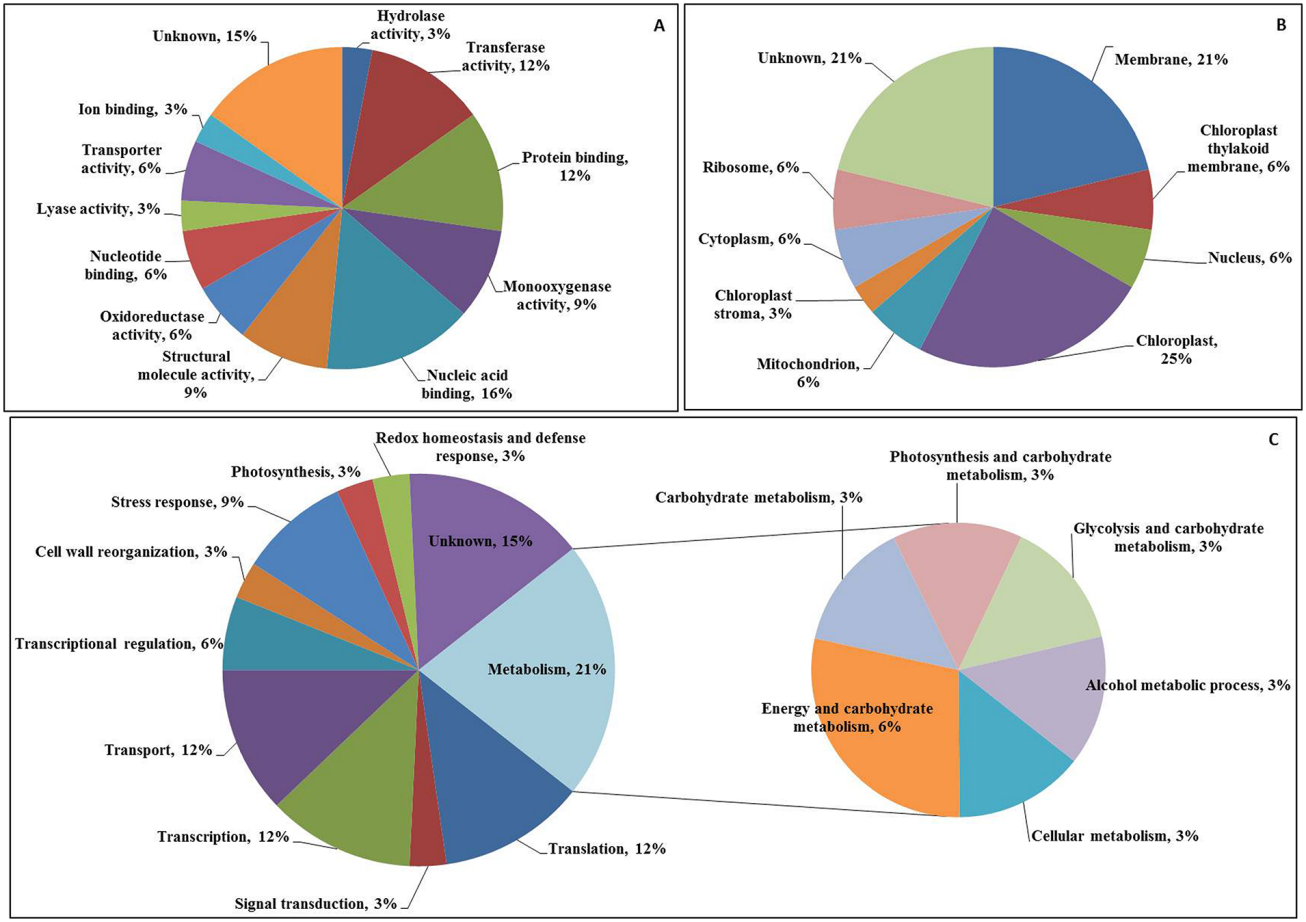
Protein encoding gene functions of 33 differentially expressed proteins identified in the leaves of *S*. *bicolor*. The frequency distribution for the identified proteins within functional categories was determined based on their molecular functions (A), cellular localization (B), and their involvement in biological processes (C). Classifications were made using iProClass databases, and the assignment of functions was based on gene ontology.

To increase our understanding of the roles of the proteins involved in Cd stress responses, the identified proteins were categorized into different groups. The 33 differentially expressed protein spots were analyzed using MALDI-TOF/TOF MS and then annotated and classified into functional categories. Gene ontology categories were assigned to all 33 proteins according to their molecular function, cellular component localization and biological processes (Fig 8A–8C). Based on their molecular functions, the proteins were classified into 12 categories. However, among all of the identified proteins, the molecular functional group corresponding to nucleic acid-binding proteins contained the largest number of differentially expressed proteins (Fig 8A), followed by the group containing transferase activity, protein-binding, structural molecule activity and monoxygenase activity-related proteins. Regarding the cellular localization, the identified proteins were classified into 9 categories. Most of the proteins were localized into chloroplasts, followed by membranes, mitochondrion, nucleus, ribosome, chloroplast thylakoid membrane and the cytoplasm (Fig 8B). The proteins were grouped into 11 biological process categories, and most of the proteins were found to be involved in metabolism, followed by translation, transcription and transport (Fig 8C).

### Putative subcellular localization model

**Fig 9:**
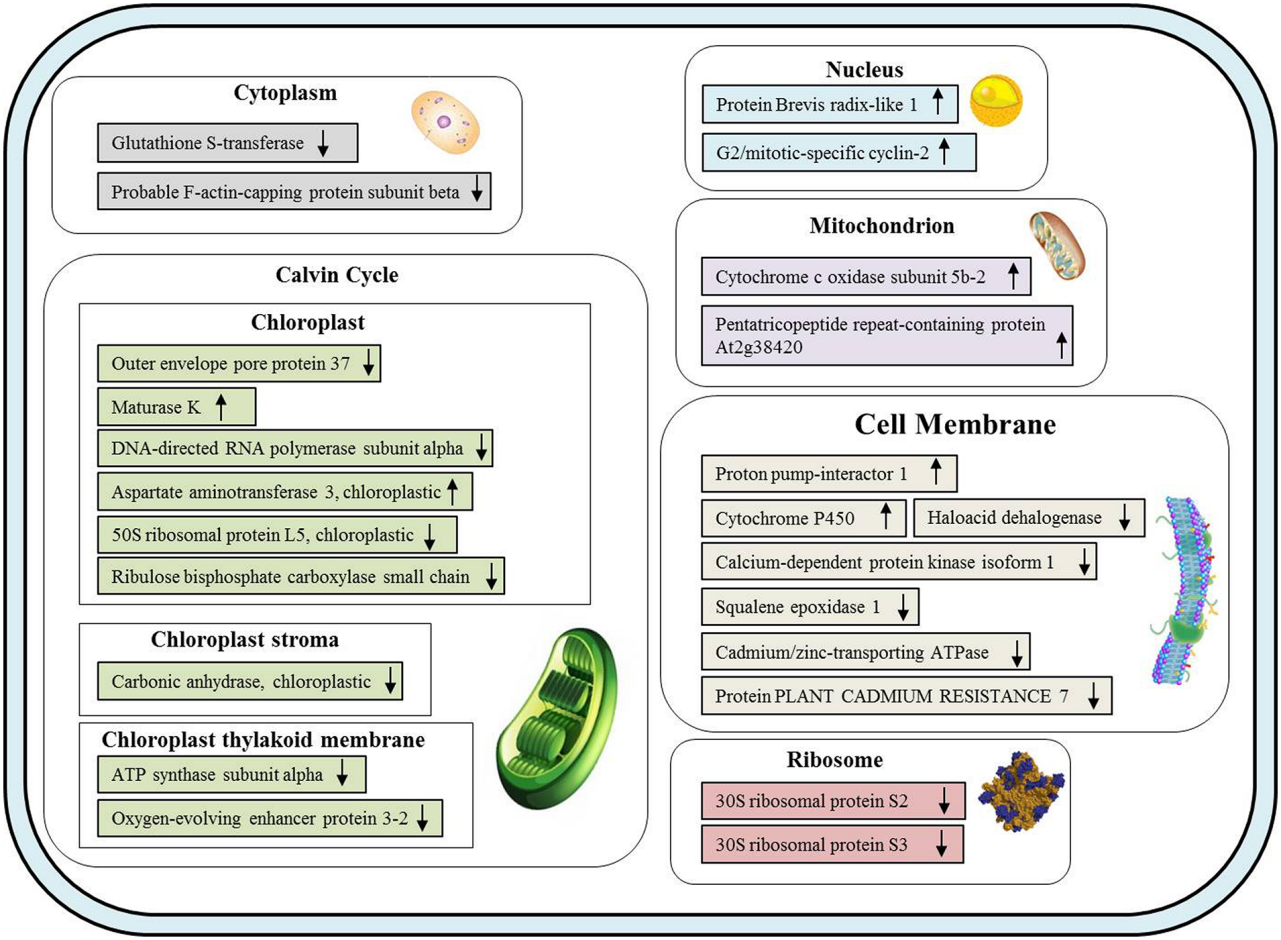
A model of the putative subcellular localization of the leaf proteins identified in *S*. *bicolor* seedlings to be affected by exposure to Cd. The Cd-responsive proteins are indicated as follows: up-regulated proteins are indicated by “↑”, and down-regulated proteins are indicated by “↓”. The proteins were categorized with regard to their localization in cellular components using iProClass databases, and the assignment of functions was based on gene ontology.

In the present study, most of the proteins identified in the leaves of sorghum seedlings exposed to Cd stress were expressed in chloroplasts and membranes (Fig 9). Among the 33 differentially expressed proteins proteins, 11 have been observed to be involved in the chloroplast (8 proteins), chloroplast thylakoid membrane (2 proteins) and chloroplast stroma (1 protein).

Next to the chloroplast, the most common localization was the membrane, with which 7 of the differentially expressed proteins are associated. These data reveal that most of the identified proteins observed to be differentially expressed were decreased in abundance under Cd stress, and the analysis of the subcellular localization of the differentially expressed proteins suggests that the chloroplast and the membrane are very sensitive to Cd stress (Fig 9).

## Discussion

### Morphological responses and changes in response to cadmium accumulation in sorghum seedlings under Cd stress

Among the many types of environmental stresses, heavy metals severely affect plant growth and development. Many studies have investigated the limiting effect of Cd on fresh and dry mass accumulation, height, root length, leaf area, and other biometric parameters in plants [52,53,54].

In the present study, to investigate the mechanism by which sorghum responds to Cd stress under hydroponic culture conditions, 10-day-old plants were transplanted and cultured in Hoagland solution containing different concentrations of Cd. This morphological investigation was focused on studying the effects of Cd on sorghum plants treated with different concentrations (0 μM, 100 μM and 150 μM CdCl_2_) of Cd. The leaves of the sorghum seedlings exhibited different accumulated effects when exposed to Cd.

A small number of studies have shown that Cd is a non-essential toxic heavy metal that causes physiological and morphological changes in plants [55]. Cadmium levels were determined in the leaves of the experimental plants in this study. A high level of accumulated Cd was observed in leaves when plants was treated with Cd. We found that the accumulated level of Cd was significantly increased in plants exposed to the highest concentration of Cd (150 μM) compared to untreated plants. In an earlier study in maize, it was shown that the highest level of Cd was obtained in plants exposed to the highest concentration of the combined effect of Cd and Zn ions [56].

Cadmium hyper accumulation has been described in many plant species in previous studies [57,58,59] including sorghum [60]. When *Phytolacca americana* plants were exposed to Cd, the leaves were severely affected by Cd, and metal ions were significantly accumulated at high concentrations of Cd [41].

### Ion concentration responses to Cd stress

When plants were exposed to a high concentration of metal, their apoplastic levels altered the aqueous and ionic thermodynamic equilibria; as a result, these plants faced hyperosmotic stress, ionic imbalance, and toxicity. In the present study, the interacting ions (Zn^2+^, Ca^2+^ and Fe^2+^) were significantly influenced by treatment with cadmium. However, an angonistic effects was observed between Cd and Zn when tobacco seedlings were subjected to Cd stress [61]. In addition, it was also previously reported that Cd significantly decrease Zn concentrations in plant tissues [62]. To get more insights into the role of Cd-induced Ca changes, the Ca ion was investigated in the present study. Ca ions were decreased significantly in response to Cd exposure. In previous work, it was revealed that Cd induced a significant reduction in the Ca content in the leaves of pea plants [63].

### Effects of absorbed Cd ions analyzed using confocal microscopy

Confocal microscopy was used to observe the distribution of the Cd that was absorbed into sorghum leaves. Dithizone is a sulfur-containing organic compound. It is a good ligand, and it forms complexes with many metals, including Cd [64]. The distribution of cadmium in sorghum leaves was investigated using dithizone staining in the present study. We found that the degree of absorption of cadmium was dose-dependant. The complex of dithizone with Cd and Pb shows as red, as previously described [65]. The fluorescence intensity of the Cd-dithizone complexes was dramatically increased in our investigation. Confocal analysis in a previous study indicated that the fluorescence intensity of similar complexes was substantially increased in the roots of wheat plants that were exposed to aluminum [49].

### Proteome changes in plants exposed to heavy metal stress

Heavy metals such as Cu, Zn, Fe, Co, Mn and Mo act as beneficial mineral element present in small quantities [66], but these metals can be toxic when present at higher concentrations [67]. Some other heavy metals that have no biological function as nutrients and that are also very toxic can restrict plant growth and development. For example, cadmium and other heavy metals such as Pb, Hg induced the production of ROS and the inactivation of enzymes [68].

The results of the present study provide information regarding the stress responses exhibited by actively growing sorghum plants placed in a controlled environment that was coupled with a hydroponic system and exposed to Cd. The present experiments were executed to investigate the molecular mechanism and, more precisely, the alterations in the proteome induced in sorghum plants under Cd stress. These proteomic data, in combination with morphological and physiological analyses provide insights into the mechanisms affected by Cd stress in hydroponically grown sorghum plants.

In the present study, proteomic analysis of the responses induced in sorghum seedling leaves by Cd stress led to the identification of 33 differentially expressed proteins. Some of the identified proteins such as glutathione S-transferase, ribulose bisphosphate carboxylase small chain [69], carbonic anhydrase, glyceraldehyde-3-phosphate dehydrogenase [41], cytochrome P450 [70] have been well characterized in response to Cd stress, while others have not been as well-studied with respect to their roles in stress responses in plants.

### Proteins involved in photosynthesis and carbohydrate metabolism

Cadmium has been shown to induce several alterations and disturbances in physiological processes [71] and to result in the dramatic inhibition of a wide range of metabolic process, including photosynthesis, carbon assimilation and photosynthetic electron transport [72]. In the present study, carbonic anhydrase (Table 1, spot number 315) was reduced by cadmium treatment. This differentially expressed protein is involved in photosynthesis. Carbonic anhydrase (CA) could alter photosystem components and consequently accelerates the deregulation of CO_2_ homeostasis, which would, in turn, promote CO_2_ depletion and the accumulation of O_2_ when leaves are subjected to high doses of Cd [28]. A significant decrease in the abundance of CA was also reported in *Phytolacca americana* [41] and in poplar [69] treated with cadmium. In addition, the down-regulation of CA was also observed in poplar plants exposed to cadmium stress [73], and two isoforms of CA were recently identified to be present at lower levels in Cd-treated plants [74]. Nevertheless, CA is thought to be an important enzyme in many biological functions, especially those that involve carboxylation/decarboxylation [75]. However, several enzymes involved in carbon fixation have also been observed to decrease in abundance, as was previously shown in a study of rice leaves [76].

### Proteins involved in glycolysis and carbohydrate metabolism

Cadmium toxicity may significantly alter the glycolytic pathway and the Cd-induced disorganization of the photosynthetic apparatus, and these effects may have an important impact on the plant’s ability to withstand this type of stress. In the present study, spot number 338 was identified as glyceraldehyde-3-phosphate dehydrogenase (GADPH). GADPH is known as an essential enzyme that catalyzes the sixth step of glycolysis, and it assists in breaking down glucose to obtain energy and carbon molecules. The key enzyme in the glycolysis process, GADPH has been observed to be increased in abundance in poplar leaves [73]. The GADPH levels were increased when *A*. *thaliana* plants were exposed to 10 μM Cd exposure [77], and its level were also increased when *A*. *thaliana* cells were exposed to different concentrations of Cd [78]. On the contrary, GADPH levels were decreased in the roots of two Cd-tolerant plants, poplar [69] and *B*. *juncea* [79], after treatment with 20 μM and 250 μM Cd respectively. GADPH was observed to be increased following treatment with both low (10 μM) and high (100 μM) levels of Cd treatment compared to its level in controls in tomato plant roots [80]. However, previous studies of the alterations observed in carbohydrate metabolism-related proteins following exposure to Cd have demonstrated contradictory findings.

Previous research has shown that the metabolic changes induced by Cd are tissue-specific. Consequently, GADPH is induced in leaf tissue but severely decreased its abundance in root tissues in poplar plants [69]. GADPH was also induced in the leaves of rice [31] and poplar plants [74] by treatment with various heavy metals. These results suggest that GADPH protein may play an active role in supplying energy to Cd-treated plants via the glycolytic pathway. Taken together, the previous reports together with the present study, indicate the changes in carbohydrate metabolism upon Cd exposure are dose dependent and plants elevate their energy consumption over energy production when it exposed to Cd stress.

### Proteins involved in energy and carbohydrate metabolism

The results of proteomic analyses have suggested that Cd has a strongly negative impact on proteins related to carbohydrate metabolism [35] and thereby, may limit the efficiency of CO_2_ fixation and reduce ATP synthesis, resulting in the inhibition of plant growth [81]. In this study, a total of three proteins involved in carbohydrate metabolism were found to be down-regulated by Cd stress, including ATP synthase subunit alpha (Table 1, spot no. 226), ribulose bisphosphate carboxylase (Rubisco) small chain (Table 1, spot no. 302) and haloacid dehalogenase (Table 1, spot no. 313). ATP synthase was observed to be markedly decreased compared to its level in the control plants. Changes in the levels of this protein may cause a decrease in ATP production in response to Cd toxicity [28].

### Cellular metabolism

One protein that was up-regulated by Cd treatment was identified as G2/mitotic-specific cyclin-2 (Table 1, spot no. 212). This protein is involved in cellular metabolism, and it was also previously found to be increased in the leaves of *A*. *paniculata* by 10 μM Cd [40], suggesting that this protein may play potential role in the regulation of cell growth.

### Proteins involved in photosynthesis

Photosynthesis is an essential metabolic process in plants, and it is also vulnerable to environmental stress. Elevated concentrations of Cd may cause deleterious effects and disturbances in transpiration and photosynthesis [82]. In our investigation, a protein called oxygen-evolving enhancer protein 3–2 (Table 1, spot no. 305) was decreased in abundance in plants exposed to Cd. Changes in the levels of this protein have also been observed in several other studies that showed that the photosystem II complex was greatly suppressed by Cd stress [37].

In addition, Cd harmfully affects protein expression related to primary carbon metabolism in plants and consequently result in the photosynthetic machinery being severely hampered by Cd stress via the degradation of major photosynthesis related proteins, such as Rubisco [37]. The amount of Rubisco (large and small subunits) was dramatically reduced by Cd stress in rice leaves [76] and algae [35]. In addition, there is little evidence for a relationship between stress and haloacid dehydrogenase (HAD). However, HAD has previously been found to be down-regulated in the marine brown algae, *Sargassum fusiforme* [83] by chronic copper stress. Taken together, the results obtained in the present study suggest that Cd may impair ATP production, limit primary carbon metabolism, hamper photosynthetic machineries and inhibit plant growth and development.

### Proteins involved in stress response

Three proteins cytochrome P450 (Table 1, spot 173), flavonoid 3', 5'-hydroxylase (Table 1, spot no. 180) and glutathione-S-transferase (GST) protein (Table 1, spot no. 228) were expressed at markedly higher levels in Cd-treated sorghum seedling leaves. Flavonoid 3', 5'-hydroxylase (F3′,5′Hs) belongs to the cytochrome P450 protein family [84]. However, cytochrome P450 (CYPs) is a stress-related protein that belong to the superfamily of proteins containing a heme cofactor. CYPs are also detoxifying enzymes that are mostly found in bacteria, archaea and eukayotes, where they are involved in protection against oxidative stress [85]. Previous evidence has indicated that a putative cytochrome P450 protein was also induced in *Physcia adscendens* [70] in response to short Cd treatments, and a pronounced inhibition of this enzyme was observed when plants were subjected to long exposure to the metal [86,87]. Microarray analysis in rice exposed to cadmium stress demonstrated that several genes encoding cytochrome P450 family proteins and other stress-related proteins were differentially expressed and that stress-related proteins were strongly induced by Cd exposure [88].

In this work, glutathione-S-transferase (GST) (Table 1, spot no. 228) was identified on 2-D gels. The level of GST was found to be increased up to 1.76-fold by Cd treatment (Table 1). GSTs, also known as ligandins, belong to a wide range of protein families [89]. GSTs catalyze the transfer of GSH to a wide variety of hydrophobic, electrophilic and cytotoxic co-substrates [90]. GST proteins are involved in sulfur and GSH metabolism. The level of GST was previously found to be increased in various plant species that were exposed to Cd stress [37,41,78]. However, three isozymes of GST identified in *Schizosaccharomyces pombe*, including GST-I, GST-II and GST-III, were found to be significantly increased by exposure to Cd stress [91]. The levels of GSTs were found to be increased in response to Cd stress, supporting the suggested roles of GST in metal detoxification [92]. Previous results also indicated that these isozymes play an active role in the detoxification of many xenobiotic compounds and they protect cells from oxidative stress [93]. In *Populus tremula*, the overall level of GST activity was higher in roots than in leaves [69]. Moreover, the up-regulation of GSTs levels in the presence of Cd^2+^ was also observed in an earlier investigation [77] in the roots of *Arabidopsis thaliana*.

The increased abundance of GSTs have been demonstrated in other heavy metal stress that involved in antioxidant defense and detoxification systems. Four GSTs (*At*GSTF2, *At*GSTF6, *At*GSTF7 and *At*GSTU19) were also identified to be significantly abundant in copper-treated *Arabidopsis thaliana* seedlings [94]. The abundance of GST enzyme has been increased markedly in both leaves and roots of cu-stressed wheat seedlings [95].

### Proteins involved in transcriptional regulation

Heavy metals limit normal plant growth and development and regulate a wide range of genes, resulting in adverse effects on many cellular responses [96]. Regulatory proteins, including pentatricopeptide repeat-containing protein (spot no. 188) and zinc finger CCCH domain-containing protein 14 (Table 1, spot no. 191) were markedly up-regulated by cadmium stress in our investigation. Pentatricopeptide repeat-containing proteins (PPR) are a family of proteins generally found in the plant kingdom, and these predicted proteins are predicted to be targeted to either mitochondria or chloroplasts [97]. Previous report have indicated that PPR protein was significantly induced in the leaves [98] and germinating seedlings of rice [99] exposed to Cd stress. However, the most interesting response to Cd exposure in rice seedling roots was a dramatic accumulation of PPR protein following exposure to low concentrations of Cd and a decrease in its abundance following exposure to a high concentration of Cd [100]. Taken together, the results obtained from these two investigations revealed that these proteins play substantial roles in RNA processing under stress conditions. An increase in the abundance of PPR protein has frequently been observed in strawberries [101] and manchurian ash [102]. These data also suggest that the PPR proteins identified in these organisms are substantially involved in the regulation of transcriptional activity.

### Proteins involved in transcription

Maturase K (Mat K) proteins (Table 1, spots 152, 183, 194) and double-stranded RNA-binding protein 1 (Table 1, spot no. 337) were identified in our study to show decreased expression during exposure to Cd stress. These proteins are involved in RNA processing, and they exhibited differing expression patterns when seedling leaves were exposed to Cd stress. In previous studies, these proteins have also been observed to be increased in abundance under stress conditions in the leaves of higher plants [103,104]. Maturase K catalyzes intron RNA binding and consequently alters gene expression at the transcriptional level [105]. However, the differential expression of these proteins has been reported in previous studies [32], suggesting that growth and development were also strongly affected by exposure to Cd. Hence, these results suggest that the changes observed in the protein expression of maturase K in plants are species- and stress-type dependent [106].

### Proteins involved in translation

In the present investigation, proteins were observed that are involved in translation, including 30S ribosomal protein S2 (Table 1, spot no. 85), 30S ribosomal protein S3 (Table 1, spot no. 213), DNA-directed RNA polymerase subunit alpha (Table 1, spot no. 203) and 50S ribosomal protein L5, chloroplastic (Table 1, spot no. 238). Our results show that two proteins (spot no. 85 and spot no. 213) were up-regulated in plants exposed to 100 μM Cd levels but down-regulated when plants were exposed to 150 μM Cd levels. Another protein (Table 1, spot no. 337) was also up-regulated when seedlings were exposed to Cd stress. However, two 30S ribosomal proteins were detected in the leaves of poplar plants [74] that were exposed to Cd stress, and the decreased abundance of these two proteins might indicate that protein synthesis and metabolism are impaired under Cd stress.

In marine cyanobacteria, six subunits of ribosomal proteins were identified, including the 30S ribosomal protein S2. The differential expression of these ribosomal proteins suggests that Cd had a substantial effect on the the character of the proteome [107]. In addition, many ribosomal proteins have been observed to be up-regulated by short-term exposure to Cd, but then later down-regulated in *Schizosaccharomyces pombe* [91]. Moreover, the down-regulation of ribosomal proteins in *Brassica napus* showed that Cd induced toxicity that affected the levels of regulation/protein synthesis-related proteins [108].

### Cell wall reorganization

Cell walls may play an active role in metal tolerance, accumulation and metal binding. Cell wall reorganization has been reported previously in heavy metal toxicity, including Cd [109]. Exposing the leaves of sorghum seedlings to cadmium stress triggered a reorganization of cell walls at both Cd concentrations, as revealed by several changes in protein levels. In the present study, cinnamyl alcohol dehydrogenase 2 (CAD) (Table 1, spot no. 237) was detected, and this protein was markedly decreased by exposure to Cd. Interestingly, several proteins related to cell wall organization were down-regulated by Cd toxicity, but, CAD was found to be particularly increased in abundance in the roots of tomato [80] and rice [110] plants by Cd stress. Hence, the obtained protein (CAD) from the present investigation, related to cell wall organization was down-regulated suggesting that the cell wall is very sensitive to Cd stress.

### Proteins involved in transport

The down-regulation of cadmium/zinc-transporting ATPase (Table 1, spot no. 290) was identified in our study. The amount of this transporter protein was significantly reduced under stress conditions in cotton [111]. A previous study in *Arabidopsis thaliana* [112] suggested that the mechanism for Cd root-to-shoot translocation relies on the activity of cadmium/zinc-transporting ATPase (HMA 2 and HMA 4). However, plant cadmium resistance 7 (Table 1, spot no. 293) was identified in the present study to be involved in the transport of proteins. However, a previous study observed that this protein mediates cadmium resistance in *Arabidopsis* [113].

### Proteins involved in redox homeostasis and defense responses

In the present study, aspartate aminotransferase 3, chloroplastic (Table 1, spot no. 336) was identified to be up-regulation by Cd stress. This protein was observed to be increased by exposure to the redox active heavy metal Cr, and has also been shown to be involved in ROX detoxification and defense responses [114].

## Conclusions

The heavy metal Cd is a global problem that seriously limits crops quality and production. Metal uptake, metal accumulation and growth responses that were associated with changes in the proteome in Cd-treated plants have not been characterized in sorghum. By examining the leaves of sorghum seedlings, the present study sheds light on the molecular mechanisms involved in Cd-tolerance in *S*. *bicolor* and suggests a more active involvement for Cd toxicity in plant morpho-physiological and molecular processes. Cadmium stress causes morpho-physiological alterations and affects the shoot and root metabolic systems. In the ionic study, the concentration of cadmium was significantly increased in the plants that were treated with the highest concentration of Cd. Proteomic analyses revealed Cd-dependant alterations in metabolic processes and in translational and transcriptional regulation-related proteins. The results from these studies were interpreted to demonstrate that Cd stress induce the general inhibition of carbon fixation, impairs ATP production and regulates protein synthesis. In addition, it is tempting to further clarify the role of GST enzymes in C_4_ plant cells in responses to Cd toxicity. These findings improve our understanding of the mechanisms that are potentially involved in plant responses to Cd stress at the protein level. Further research is required for a better understanding of cellular and molecular responses to cadmium.
